# FPGA Implementation for Real-Time Background Subtraction Based on Horprasert Model

**DOI:** 10.3390/s120100585

**Published:** 2012-01-05

**Authors:** Rafael Rodriguez-Gomez, Enrique J. Fernandez-Sanchez, Javier Diaz, Eduardo Ros

**Affiliations:** Department of Computer Architecture and Technology, ETS Computer Engineering and Telecommunications, University of Granada, C/ Periodista Daniel Saucedo s/n, E18071 Granada, Spain; E-Mails: rrodriguez@atc.ugr.es (R.R.-G.); jdiaz@atc.ugr.es (J.D.); eduardo@atc.ugr.es (E.R.)

**Keywords:** real time image processing, reconfigurable architectures, FPGAs, performance analysis, video surveillance

## Abstract

Background subtraction is considered the first processing stage in video surveillance systems, and consists of determining objects in movement in a scene captured by a static camera. It is an intensive task with a high computational cost. This work proposes an embedded novel architecture on FPGA which is able to extract the background on resource-limited environments and offers low degradation (produced because of the hardware-friendly model modification). In addition, the original model is extended in order to detect shadows and improve the quality of the segmentation of the moving objects. We have analyzed the resource consumption and performance in Spartan3 Xilinx FPGAs and compared to others works available on the literature, showing that the current architecture is a good trade-off in terms of accuracy, performance and resources utilization. With less than a 65% of the resources utilization of a XC3SD3400 Spartan-3A low-cost family FPGA, the system achieves a frequency of 66.5 MHz reaching 32.8 fps with resolution 1,024 × 1,024 pixels, and an estimated power consumption of 5.76 W.

## 1. Introduction

Extracting background from a video sequence is a required feature for many applications related to video surveillance: vehicle traffic control, intruders’ detection, suspicious objects, *etc*. The most usual approach to segment moving objects is known as background subtraction, and is considered a key first stage in video surveillance systems. This technique consists of building a reference model which represents the static background of the scene during a certain period of time. Multiple factors and events may affect the scene, making this first background subtraction a non-trivial task: sudden and gradual illumination changes, presence of shadows, or background repetitive movements (such as waving trees), among many others.

There are different methods described in the literature in order to obtain this background model for a scene captured by a still camera: simple models for static backgrounds [1,2], or methods capable to deal with periodic and repetitive movements, *i.e.*, waving trees or escalators such as MOG (Mixture of Gaussians) [3,4], Bayesian decision rules [5], Codebook-based model [6], or Component Analysis (PCA and ICA) [7,8]. In spite of the differences between existing algorithms, background subtraction techniques are computationally expensive in general, especially when they are considered only the first stage in a multi-level video analytics system. For that reason, efficient implementation is key to the development of real-time video surveillance systems. In the framework of embedded systems implementations, characterized by power consumption and real-time constraints, several of these techniques have been implemented using FPGAs [8–11] or DSPs [12]. There also are other real-time approaches using GPUs [13,14]. In the case of embedded systems, commodity processor implementations are not usually utilized although latest devices, such as Intel Atom, could soon address this market. In this contribution, we focus on a type of implementation that fits a distributed architecture.

Oliveira *et al*. [11] introduce an FPGA implementation for the Horprasert algorithm, although the throughput reached by this approach is fairly low. Jiang *et al*. [10] present a compression scheme for Mixture of Gaussians model [3] which allows reaching a high frame rate. However, this approach is not explained in detail, and no results are shown with regards to accuracy or power consumption. Appiah *et al*. [9] propose an implementation based on a simplified MOG, which offers fairly good throughput and acceptable accuracy. Bravo *et al*. [8] propose an FPGA implementation based on Principal Component Analysis (PCA), reaching a good throughput and specifying the resource consumption. Nevertheless, there are no data about accuracy with a standardized dataset. Carr [13] and Vu Pham *et al*. [14] present GPU implementations based on MOG, with very different results in performance. Despite the fact that the approach described in [14] has a higher frame rate than any of the other mentioned hardware implementations, being a GPU implementation is an impediment for embedded systems and low power constraints. Strictly speaking, there are new GPU families oriented to embedded devices that could solve that problem. However, the performance of these GPU families is considerably lower than the ones of GPU used on standard PCs or laptops.

In our approach, we propose an FPGA architecture based on the method described by Horprasert [1,2], with the extension that allows for shadow detection [15]. Thus, the use of FPGAs is justified by requirements of scalability, size and low power consumption which are key features that other technologies are not able to achieve. The Horprasert method has been selected since it requires less memory to store the model while keeping fairly good accuracy, hence being more suitable for implementation in low cost FPGAs [11]. This algorithm builds a static background model, which means that the model is obtained at an initial training phase. There are other methods which build dynamic background models [3,6], which can adapt themselves to changes in the scene. The main difference between these models, as far as required hardware resources are concerned, is that the latter have much higher memory consumption requiring external memory with an important bandwidth. Furthermore, the shadow detection capabilities increase the accuracy of the object shape detection, which helps to achieve a better object classification and reduces the errors due to shadows artifacts.

Therefore, the main contribution of this paper is the implementation of a background subtraction model based on Horprasert and extended to allow shadow management in FPGA. This added feature as well as a careful design, keeping appropriate bit depth in different variables computed with fixed point arithmetics, enhance the accuracy compared to previous hardware-based approaches described in the literature whilst maintaining good throughput (more than 35 times faster than the previous Horprasert-based approach [11]). This high data throughput is achieved through an intensively parallel design. This approach targets low end embedded devices. In order to properly evaluate the presented implementation, a comparison with other approaches is included, which is something rarely covered in the literature related to hardware implementation of background subtraction models. This comparative study integrates estimations related to the implemented model accuracy and also data throughput to better evaluate the proposed system in the framework of real-time approaches. To the best of our knowledge this complete comparative study including computational performance in terms of accuracy and efficiency has not been reported before and allows comparisons with future alternatives in this application field.

The proposed architecture has been designed with the development environment for System-on-Chip (SoC) design, EDK of Xilinx [16], and includes the Microblaze processor, which will be used to build the reference background model and can be used for updating over time. The next stages of subtraction and pixel by pixel classification will be performed by a specific hardware module using fixed-point operations in order to keep real-time performance from up to four still cameras simultaneously.

The paper is organized as follows. In Section 2 we briefly describe the background model by Horprasert *et al*. [1], including the notation required in order to be able to follow the rest of the paper. Section 3 shows the developed hardware architecture, including a study of the fixed point arithmetic, the background subtraction stage and the technique for morphological filtering on FPGA. In Section 4 results are shown and analyzed, regarding system performance and comparison with other hardware approaches as well as accuracy results obtained by the algorithm. Finally, conclusions and future work are presented in Section 5.

## 2. Background Subtraction Method

As previously mentioned, our implementation is based on the algorithm proposed by Horprasert *et al*. [1]. This algorithm basically obtains a reference image to model the background of the scene so that it can perform automatic threshold selection, subtraction operation and, finally, pixel-wise classification.

### 2.1. Background Model

In order to build a reference image which represents the background, a number *N* of images will be used, whose color space is given in RGB. Each pixel *< i >* from the image is modeled by a 4-tuple *< E_i_*, *S_i_*, *a_i_*, *b_i_* *>*, where each element is defined as follows:

- *E_i_* the expected color value, defined as *E_i_* = [*μ_R_*(*i*)*, μ_G_*(*i*)*, μ_B_*(*i*)], with *μ_R_*(*i*)*, μ_G_*(*i*)*, μ_B_*(*i*) being the arithmetic means of each color channel for pixel *i*.
- *S*_*i*_ the value of the color standard deviation for each channel, defined as *S_i_* = [*σ_R_*(*i*)*, σ_G_*(*i*)*, σ_B_*(*i*)].
- *a_i_* the variation of the brightness distortion, computed as the root mean square (RMS) of the brightness distortion *α_i_*, given by Equation (1).
  (1) $$\alpha_{i}=\frac{\frac{I_{R}(i)\mu_{R}(i)}{\sigma_{R}^{2}(i)}+\frac{I_{G}(i)\mu_{G}(i)}{\sigma_{G}^{2}(i)}+\frac{I_{B}(i)\mu_{B}(i)}{\sigma_{B}^{2}(i)}}{{\left(\frac{\mu_{R}(i)}{\sigma_{R}(i)}\right)}^{2}+{\left(\frac{\mu_{G}(i)}{\sigma_{G}(i)}\right)}^{2}+{\left(\frac{\mu_{B}(i)}{\sigma_{B}(i)}\right)}^{2}}$$
- *b_i_* the variation of chromaticity distortion, the RMS of the chromaticity distortion *CD_i_*, which is described in Equation (2).
  (2) $${\mathrm{CD}}_{i}=\sqrt{{\left(\frac{I_{R}(i)-\alpha_{i}\mu_{R}(i)}{\sigma_{R}(i)}\right)}^{2}+{\left(\frac{I_{G}(i)-\alpha_{i}\mu_{G}(i)}{\sigma_{G}(i)}\right)}^{2}+{\left(\frac{I_{B}(i)-\alpha_{i}\mu_{B}(i)}{\sigma_{B}(i)}\right)}^{2}}$$

More detailed information about how the background model is built can be found in [1].

### 2.2. Subtraction Operation and Classification

In this stage, the difference between the background model and the current image is evaluated. This difference consists of two components: brightness distortion *α_i_* and chromaticity distortion *CD_i_*. In order to use a single threshold for all pixels, it is necessary to normalize *α_i_* and *CD_i_* as follows:

(3) $${\hat{\alpha }}_{i}=\frac{\alpha_{i}-1}{a_{i}}$$

(4) $${\hat{\mathrm{CD}}}_{i}=\frac{{\mathrm{CD}}_{i}}{b_{i}}$$

**Figure 1.**
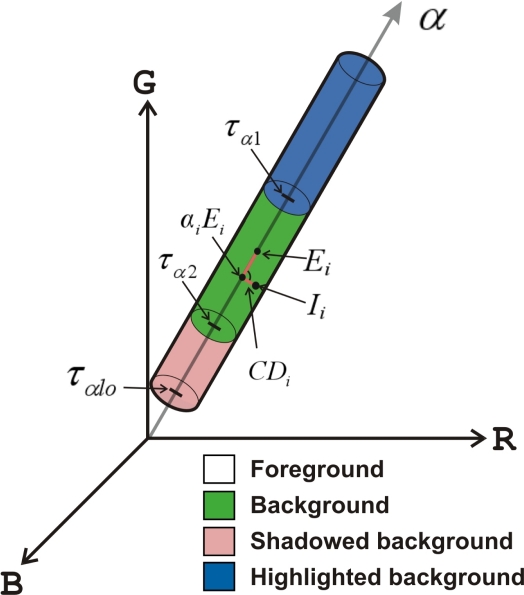
Graphic representation of the model used to classify the pixels in the categories. This model is oriented to shadow and highlights detection, taking into account chromaticity lines as well as brightness changes.

After the normalization of brightness and chromaticity distortions, the given pixel can be classified into one of the four categories, *i.e.*, *Background*, *Shadowed background*, *Highlighted background* and *Foreground*, by the decision procedure described in Equation (5), that is the analytical representation derived from the model presented in Figure 1.

(5) $$C(i)=\{\begin{array}{ll} \text{Foreground}: & {\hat{\mathrm{CD}}}_{i}>\tau_{\mathrm{CD},}\;\text{or}\;{\hat{\alpha }}_{i}<\tau_{\alpha \mathrm{lo}}\;\text{else}\\ \text{Background}: & {\hat{\alpha }}_{i}>\tau_{\alpha 1,}\;\text{and}\;{\hat{\alpha }}_{i}\geq \tau_{\alpha 2}\;\text{else}\\ \text{Shadowed background}: & {\hat{\alpha }}_{i}<0,\;\text{else}\\ \text{Highlighted background}: & \text{otherwise} \end{array}$$

The thresholds *τ_CD_*, *τ_α_*_1_, *τ_α_*_2_ are automatically selected from the information obtained during the training stage, as explained in [1]. *τ_αlo_* is a lower bound used to avoid misclassification of dark pixels.

This approach to shadow detection is considered a Statistical non-parametric (SNP) method [15], what means that the approach uses probabilistic functions to describe the class membership, and it is non-parametric since the thresholds are automatically determined by means of a statistical learning procedure. As previously mentioned, this stage is performed by a hardware module. For that reason, several modifications have been made in order to reduce the hardware complexity of the architecture. These simplifications towards a hardware friendly model generate some degradation on the original model’s quality that will be evaluated in subsequent sections. These modifications will be described in Section 3.1.

## 3. Hardware Architecture

An optimized hardware architecture has been developed using novel ideas that allow for a high degree of algorithm tuning for optimized digital hardware implementation. They can be summarized as follows:

- Hardware/software co-design. The use of a mixed hardware/software architecture allows us to share its resources to solve many algorithm stages as the ones related with communication, system initialization, basic control, system debugging, *etc*. . . . It is not necessary to develop custom datapaths for these stages because no critical real-time restrictions are imposed to them. This permits to reduce hardware resources, to extend the system flexibility and to significantly reduce development time.
- Superscalar and pipelined architecture. Multiple functional units run in parallel to adapt the intrinsic algorithm parallelism. The whole implementation has been carefully pipelined in order to increase the throughput. These strategies allow us to keep the pixel-rate very high and to achieve a significant performance.
- Adaptable fixed-point arithmetics. The bit-width of the different processing stages has been tuned according to the accuracy requirements of each processing element. This approach is very different from the one used on many DSPs or digital hardware implementations that has a basic bit-width for all the processing stages. Our approach allows us to keep resources always tuned to the required accuracy at the cost of increasing the complexity of the system design. Hopefully, the use of high-level description languages helps to reduce the development time and make this option feasible with acceptable design time.
- Proper utilization of the right level of abstraction for description of the different algorithm modules. The processing stages mainly require a DSP-based design flow which is well described using high-level description languages (as provided by ImpulseC [17]) whilst basic controllers such as the ones required for memory interfaces or low level communications are better described in RTL (for instance using VHDL or Verilog). In addition, sequential operations such as the ones required to build communication packages are well described by software code. Our implementation uses different descriptions based on the previous considerations. This enables to get the maximum output out of each description level in terms of performance or development time.

As it could be understood from the previous sentences, the advantage of our implementation relies on the combination of the latest design methodologies, seldom addressed together in the same design. The drawback of this novel approach is that it requires a high degree of competences at many different design levels, languages and tools. Nevertheless, the advantage is that it allows highly optimized designs that completely fit the target application.

In order to address this implementation, we use EDK (Embedded Developer’s Kit) of Xilinx Inc. [16]. The EDK environment facilitates the design of complex and completely modular SoC architectures able to support embedded microprocessors (MicroBlaze, PowerPC, . . .), peripheral and memory controllers (Ethernet, DDR2, ZBT, . . .), and interconnecting buses (PLB, NPI, MCH, FSL, . . .), whilst IP cores for specific processing can be designed using HDL languages through the ISE tool. As board we use the ViSmart4 video processing board from Seven Solutions [18], including: two Xilinx XC3SD3400aFG676 FPGAs, two 256 MB DDR2 DIMM memory modules, four independent analog video inputs, two gigabit ethernet connections, 485 connection, a 3G connection module, a 64 MB Flash memory, and two 1 MB *×* 36 bits ZBT memories. In our case, we have only used one of the FPGAs included in the ViSmart board, the other one is used for communication purposes.

**Figure 2.**
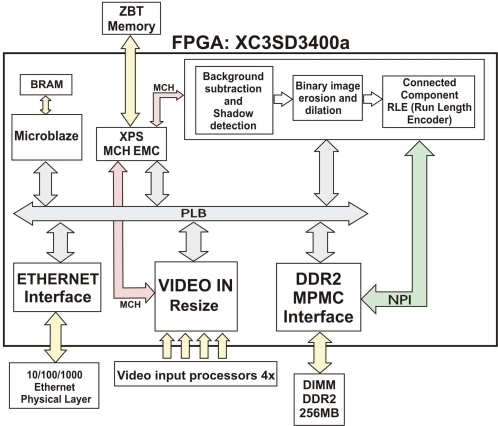
Scheme of the complete architecture and connections between modules, peripherals, memory and processor.

This architecture consists of several modules and interconnect buses, as shown in Figure 2. Processing modules, peripherals and a Microblaze processor are connected to a PLB bus. The VIDEOIN module captures images from four independent analog inputs and stores them in a ZBT SSRAM external memory, through the MCH port of the XPS MCH EMC memory interface module. Through the PLB bus, Microblaze has access to: memory regions (ZBT or DDR2), configuration registers of the peripherals and the ethernet interface for data and image sending/receiving. The Background subtraction, shadow detection and blob detection (erosion, dilation and RLE) module performs an intensive processing on the pixels of each image in order to separate foreground and background, and then proceeds to the blob extraction of the different objects. This module uses ImpulseC [17] in order to develop a DSP-based design flow system. The Microblaze processor is programmed in C/C++ for initialization and communications tasks, and the rest of peripherals are described in VHDL. The MPMC module (DDR2 memory controller) offers an easy access to the external DDR2 memory, which stores the background model. This memory offers efficient high bandwidth access, thus providing a feasible use for applications requiring real-time processing.

**Figure 3.**
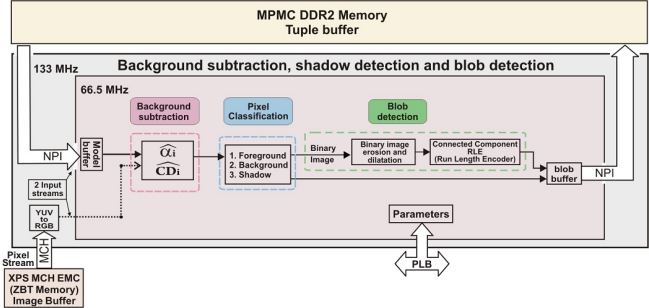
Simplified datapath architecture for background subtraction and blob detection core. The IP core can process streams from up to four cameras.

Figure 3 shows a basic scheme of the proposed architecture for the IP core that performs the background subtraction, pixel classification and blob detection processing stages. This architecture consists of a pipelined structure divided into several basic stages which work in parallel. In addition, it is controlled by means of the embedded Microblaze processor. Before getting into the details of this architecture, it is important to note that memory has a key role in the system performance and requires an efficient memory accessing scheme. This has motivated the use of high performance multiport memory controllers (Xilinx MPMC for DDR2) as well as very specific and optimized memory ports (NPI).

### 3.1. Model Modifications Towards a Hardware-Friendly Implementation

The foreground/background segmentation is executed by a hardware module with an independent access to the memory, where the current image and the background model are stored. Considerable reduction of the hardware complexity of the architecture is achieved through precalculating and storing several constants during the training stage and avoiding division operations by substituting them for multiplications, which require less hardware resources. In the case of brightness distortion *α_i_*, these constants are computed according to Equation (6):

(6) $$\begin{array}{l} A_{i}={\left(\frac{\mu_{R}(i)}{\sigma_{R}(i)}\right)}^{2}+{\left(\frac{\mu_{G}(i)}{\sigma_{G}(i)}\right)}^{2}+{\left(\frac{\mu_{B}(i)}{\sigma_{B}(i)}\right)}^{2}\\ B_{i}=\left(\frac{\mu_{R}(i)}{A_{i}\sigma_{R}^{2}(i)}\right)\\ C_{i}=\left(\frac{\mu_{G}(i)}{A_{i}\sigma_{G}^{2}(i)}\right)\\ D_{i}=\left(\frac{\mu_{B}(i)}{A_{i}\sigma_{B}^{2}(i)}\right) \end{array}$$

The brightness distortion *α_i_* will remain as in Equation (7), making use of the constants *B_i_*, *C_i_*, *D_i_*.

(7) $$\alpha_{i}=B_{i}I_{R}(i)+C_{i}I_{G}(i)+D_{i}I_{B}(i)$$

In order to remove the divisions in the computation of the chromaticity distortion *CD_i_*, we store (*S_i_*)*^−^*^1^, (*a_i_*)*^−^*^1^ and (*b_i_*)*^−^*^1^ instead of *S_i_*, *a_i_* and *b_i_*. Besides, the training stage is done with *N* = 128 images to facilitate the computation of the mean, standard deviation and root mean square, avoiding divisions. Previously, the model had a 4-tuple for each pixel, composed by *< E_i_*, *S_i_*, *a_i_*, *b_i_* *>*, whereas now a 7-tuple will have to be stored*< E_i_*, *B_i_*, *C_i_*, *D_i_*, (*S_i_*)*^−^*^1^, (*a_i_*)*^−^*^1^, (*b_i_*)*^−^*^1^ *>*. The hardware complexity has been reduced considerably, but at the cost of increasing memory consumption, since now we also have to store the constants *B_i_*, *C_i_* and *D_i_*.

The software implementation has been developed using double floating-point representation. This allows reaching a higher degree of accuracy at the expense of a worse performance on embedded devices. In order to develop a hardware implementation on FPGA with constrained resources, a fixed-point representation is usually employed since it adjusts itself better to the type of available resources, although a detailed study is required in order to optimize the trade-off between accuracy and hardware consumption. It is important to take into account that an insufficient number of bits may lead to inaccurate results with high quantification noise. On the contrary, the use of too many bits can increase the hardware resources consumption, making the system implementation on a moderate cost FPGA unfeasible.

In order to determine the appropriate number of bits for the fractional part of variables *< E_i_*, *B_i_*, *C_i_*, *D_i_*, (*S_i_*)*^−^*^1^, (*a_i_*)*^−^*^1^, (*b_i_*)*^−^*^1^ *>* which represent our background model, we have measured the error between the results obtained with different bit-width configurations and the results obtained with a double-floating-point representation. In order to perform this comparison, we have used the Wallflower test database [19]. Since we are dealing with a two-class classification problem (foreground and background), comparison measures will be given by the total of false positive and negative (FP and FN) in a certain frame of each sequence from Wallflower test. Each of the mentioned variables intervenes in a stage of the computation of *α̂_i_* and ${\hat{\mathrm{CD}}}_{i}$. In order to simplify the procurement of the appropriate number of bits for each variable, we have grouped these variables depending on the stage in which they are used to study the bit-width changes jointly.

**Figure 4.**
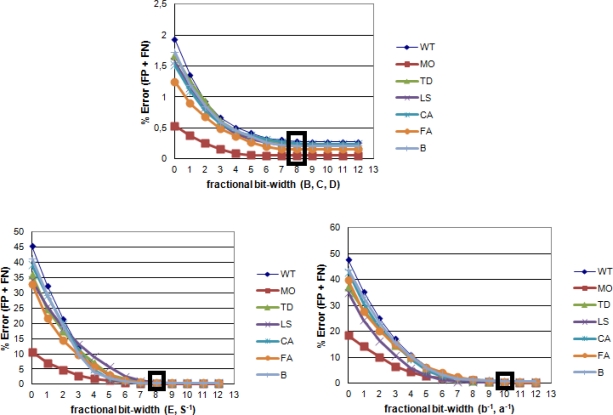
Percentage of errors between the floating point and fixed point versions, considering different bit-width of the fractional parts of the variables involved in the background subtraction. Each graph shows variables involved in the same stage of computation, and each series corresponds with a Wallflower test sequence: *WT—Waving Trees; MO—Moved Object; TD—Time of Day; LS—Light Switch; CA—Camouflage; FA—Foreground Aperture; B—Bootstrapping*.

Figure 4 displays the percentage of errors between the floating point and fixed point implementations, for each one of these groups of variables. For the first group of variables *< B_i_*, *C_i_*, *D_i_* *>* which are used in the computation of *α_i_*, an ideal representation would be 8 bits for the fractional part and 18 bits for the integer part. The second group of variables, consisting of *< E_i_*, (*S_i_*)*^−^*^1^ *>*, which is used in the computation of *CD_i_*, would have an ideal representation of 8 bits for the fractional part and 8 bits for the integer part. Finally, regarding the group *<* (*a_i_*)*^−^*^1^, (*b_i_*)*^−^*^1^ *>* which is used in the final computation of *α̂_i_* and ${\hat{\mathrm{CD}}}_{i}$, an ideal representation would be 10 bits for the fractional part and 8 for the integer part (Table 1 and Figure 4).

Each data structure has a different bit-width which is optimized to the type of performed operations (multiplications, summations, subtractions, *etc*.). This is not a common approach in the literature because of the increase in the complexity of the design stage but allows tuning resources and accuracy of the system in a much finer way.

Once the bit-width of the fractional part of each variable has been established, we have evaluated the degradation of our complete design due to quantization errors. These results are shown in Section 4.3.

### 3.2. Background Subtraction and Pixel Classification

The following stages of processing, including background subtraction, pixel classification, morphological filtering for erosion and dilation, and connected component detection, have been described using ImpulseC [17].

At the system initialization stage, after acquiring 128 images per camera and storing them into memory, the background model of the scene can be constructed. As commented before, our system is able to work with up to four camera streams. Each video sequence from each camera is independent from the others and has its own background model. Taking into account that the background model is built only in the beginning, we have considered more appropriate for the construction to be made by a software application running in the Microblaze processor, since during this stage there are not real-time requirements. Data of the tuple *< E_i_*, *B_i_*, *C_i_*, *D_i_*, (*S_i_*)*^−^*^1^, (*a_i_*)*^−^*^1^, (*b_i_*)*^−^*^1^ *>* are computed in floating point notation by Microblaze, although they are stored in memory in fixed point to be used by the hardware module. The size of a fixed point tuple will determine the required memory space for the background model, in our case *< E_i_* (48 bits), *B_i_* (26 bits), *C_i_* (26 bits), *D_i_* (26 bits), (*S_i_*)*^−^*^1^ (48 bits), (*a_i_*)*^−^*^1^ (18 bits), (*b_i_*)*^−^*^1^ (18 bits)*>* in total 210 bits. In order to maintain a background model for a 1,024 *×* 1,024 image, we will need 26.25 MB. This is affordable for the current resources available on many FPGA platforms and validated the feasibility of our current architecture. Note that this process could be triggered at any time if we determine that the background has been modified, for instance due to important lighting conditions changes or because of the appearance of new objects in the scene.

**Figure 5.**
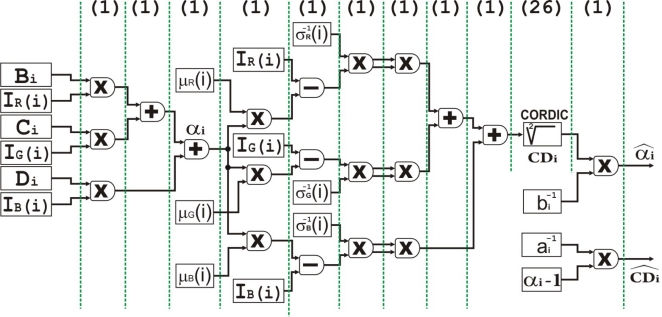
Fine grain pipelined datapath for background subtraction stage. The number of clock cycles is indicated on top. The different operations are indicated by × sign (multiplication) and + sign (addition), registers by rectangles while routing paths are indicated by arrows.

The first stage executed by the IP core is background subtraction. Once the Microblaze processor has built the background model, the subtraction and pixel-wise classification stages, shown in Figure 3, will be performed by an IP core connected to the MPMC interface by means of the NPI port. These critical interfaces with external devices are described with VHDL language. This module has been designed with the high level of abstraction hardware description language *ImpulseC, IMPULSEC* and has two input streams (background model *BG*(*i*) and current image *I*(*i*)) and one output stream (binary mask *M*(*i*)). Figure 5 shows in more detail the fine-grain pipelined datapath for this hardware module. The multipliers used are optimized with embedded resources (DSP48) of the Spartan3 DSP FPGA. In order to compute the square root of ${\mathrm{CD}}_{i}^{2}$, we have used a “Xilinx IP core” generated with the tool Core Generator and based on the CORDIC algorithm (Parallel Architectural Configuration). The total number of stages (latency) of each scalar unit is 36, with a data throughput of 1 data per clock cycle. It is remarkable that the parallel CORDIC core has 26 pipelined stages in total, being able to produce a new output data each cycle.

### 3.3. Blob Detection

**Figure 6.**
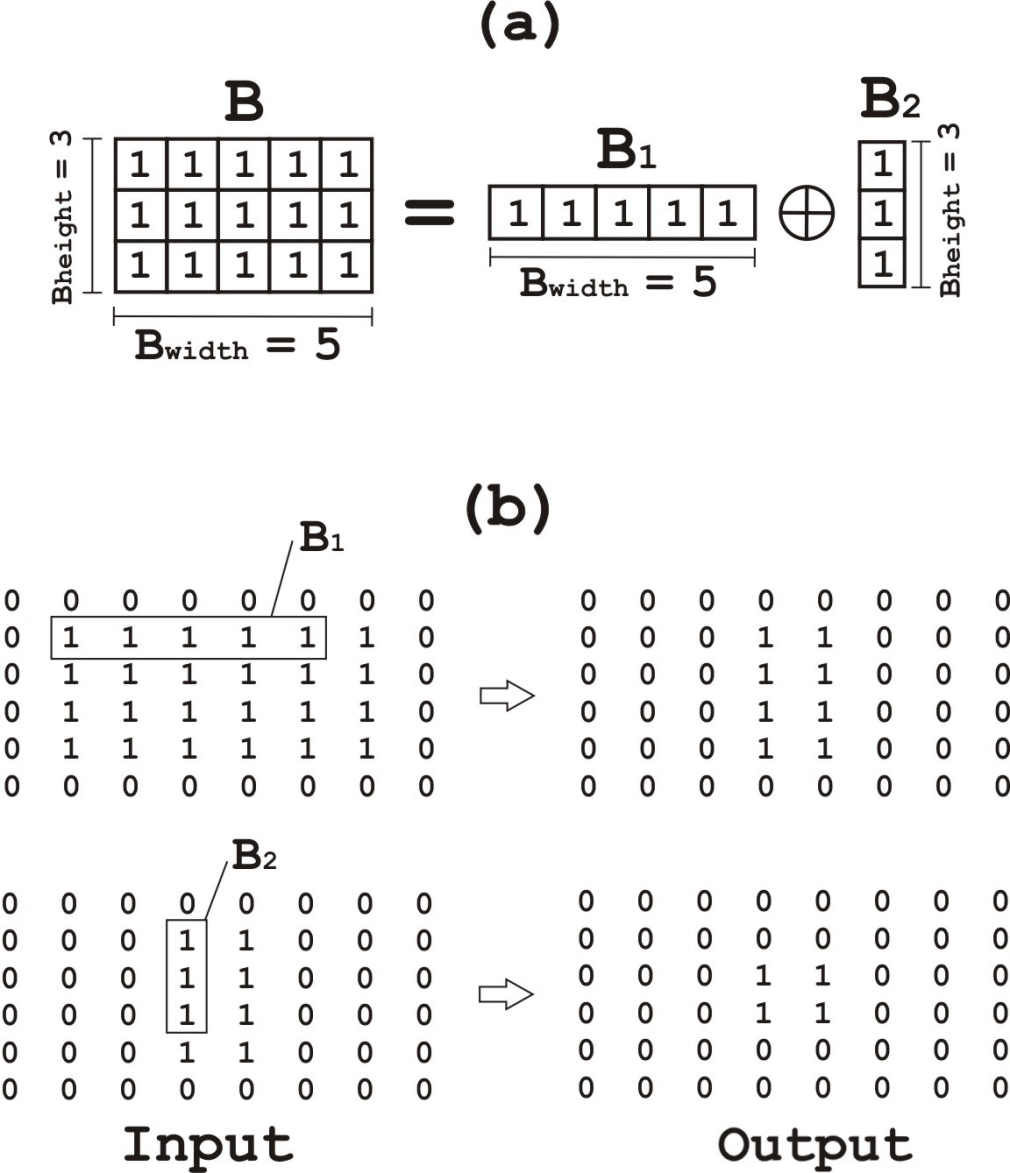
**(a)** Decomposition of structuring element *B* = *B*1 *⊕ B*2; **(b)** Input and output to decomposition windows *B*1 and *B*2.

After conducting background subtraction, the system generates a binary mask image in which 0 and 1 represent background and foreground respectively. In theory, moving objects from the image should be detected as independent elements in the binary mask image; however, this binary mask image might include noise and individual objects decomposed in multiple units; this is due to the moving object having some similar colors to the background. In order to remove noise and connect the decomposed objects again, morphological operations (erosion–dilation) are applied to the binary mask image, making use of the architecture described by Hedberg *et al*. [20], where a low complexity architecture using Structuring Element Decomposition is proposed. This is not part of the Horprasert model but we have adopted this extension in order to increase the system accuracy at a low cost. The proposed system for binary E&D requires low consumption of hardware resources (logic and internal memory). Erosion and dilation are represented by (*A ⊖ B*) and (*A ⊕ B*) respectively, where *A* is the input binary image and *B* is the structuring element (SE). *B* has some limitations in the proposed architecture: it must have a rectangular shape (any length and width is allowed) and it may only contain ones. Thus, we get *B* = (*B*1 *⊕ B*2), and *B* will be decomposed in smaller SEs (Figure 6), therefore, *A ⊕ B* = *A ⊕* (*B*1 *⊕ B*2) = (*A ⊕ B*1) *⊕ B*2. As conclusions, *B* is decomposed into two different 1-D operations, which is the key task that allows simplifying the hardware architecture of the system and reducing resources utilization.

As we can see in detail in Hedberg’s model [20], if the SE is both reflection invariant (*i.e.*, *B* = *B̂*) and decomposable, then

(8) $$\begin{array}{l} A⊕B={\left((A^{\prime }⊖B1)⊖B2\right)}^{\prime }\\ A⊖B=(A⊖B1)⊖B2 \end{array}$$

where*′* is bit inversion.

The total number of comparisons conducted for each output is equal to the number of ones in *B*; however, when it comes to a decomposed SE, the number of comparisons is reduced to the sum of ones in *B*1 and *B*2. Therefore, if *B* has 15 elements (3 × 5), the result of *B*1 + *B*2 is 8 elements (3 + 5), the number of comparisons per output is decreased from 15 to 8.

**Figure 7.**
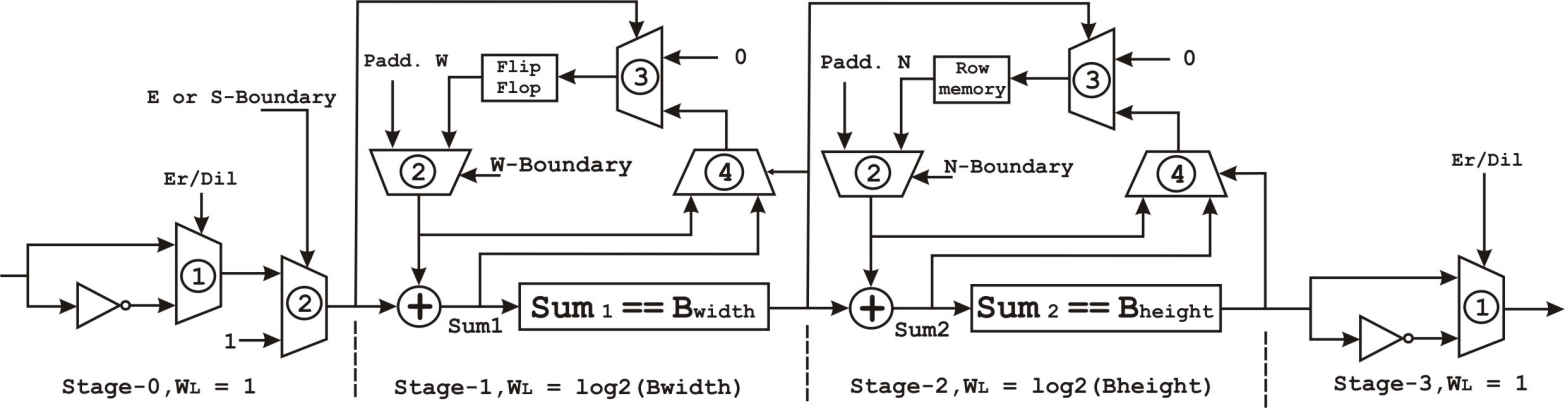
Architecture of the datapath within the erosion and dilation unit together with the wordlenghts (WL) in each stage. The input and output sections, stage-0 and 3, have a single bit wordlength. In stages 1 and 2, the wordlengths are *log*_2_(*Bwidth*) and *log*_2_(*Bheight*), respectively. The wordlength determines the total size of the memory required to perform dilation and erosion.

The proposed architecture for the morphological operations is based on Equation (8). Figure 7 shows the final architecture of the datapath. The same hardware can be used to perform both operations (erosion and dilation) on a decomposed SE. When combining this with decomposition, the summation can be broken up into two stages, where the first stage, stage-1, compares the number of ones under *B*1 to the width of *B*1 and the second stage, stage-2, compares the number of ones under *B*2 in the result from stage-1, to the height of *B*2. In order to perform dilation, the input *A* and the result are inverted using the multiplexers indicated with number 1.

Due to the rectangular structure of the kernel SE, the erosion can be performed as an addition followed by a comparison. In the first stage, each bit from *A* that overlaps with the current position of *B*1 would be added and the total result is compared against the width of *B*. If the addition is equal to the width of *B* the result will be one, otherwise it is set to zero. This addition is stored in the stage-1 flip-flop. When the input is 1, the total addition is increased and, on the contrary, the sum is reset to zero using the multiplexor marked with number 3. Each time the total addition from stage-1 matches the width of *B*, its output becomes 1, therefore the multiplexor marked as 4 will insert into the flip-flop the value *Bwidth −* 1 to be compared with the next input bit. The same operating structure is used in stage-2, storing the number of consecutive overlaps from the first stage for each column in *A*. Finally, an external controller will set the north and west padding values and produce the control signals *W − boundary*, *N − boundary*, and *E* or *S − boundary*.

We have included this architecture in our circuit in order to perform the opening (erosion–dilation) and closing (dilation–erosion) operations on the binary mask image. Each E&D operation consists of four stages; therefore, in order to perform both the opening and closing operations we will have 16 pipelined stages overall, with a data throughput or rate of 1 result per clock cycle.

Finally, once the morphological operations have been conducted, the binary mask image contains groups of connected pixels representing different relevant objects (blobs). In order to separate and differentiate these groups, we will use the algorithm described in Appiah *et al*. [21], which associates each pixel to one label placing it into a particular group.

This architecture is divided into different stages running in parallel. First, at the stage PixelToRuns(T), the pixels in each of the binary mask image rows are represented using a run-length encoding. Each run has the values *ID*, *EQ*, *s*, *e*, *r*, where *ID* is the identity number of the run, *EQ* is the equivalence value, *s* the *x*-offset of the start pixel, *e* the *x*-offset of the end pixel, and *r* the row. The run-length encoded format is a compact representation which allows for an efficient use of the FPGA internal memory.

The second stage, InitLabelling(runs), involves initial labelling and propagation of labels, for each run. All the runs are scanned, assigning provisional labels which propagate to any adjacent runs on the row below; runs one row below are scanned for an overlap. An overlapping run in 4-adjacency (*si ≤ ej* and *ei ≥ sj*) or 8-adjacency (*si ≤ ej* + 1 and *ei* + 1 *≥* *sj*) is assigned with the identity *ID_i_*, if and only if *ID_j_* is unassigned. If there is a conflict (if an overlapping run has assigned *ID_j_*), the equivalence of run *i*, *EQ_i_* is set to *ID_j_*.

**Figure 8.**
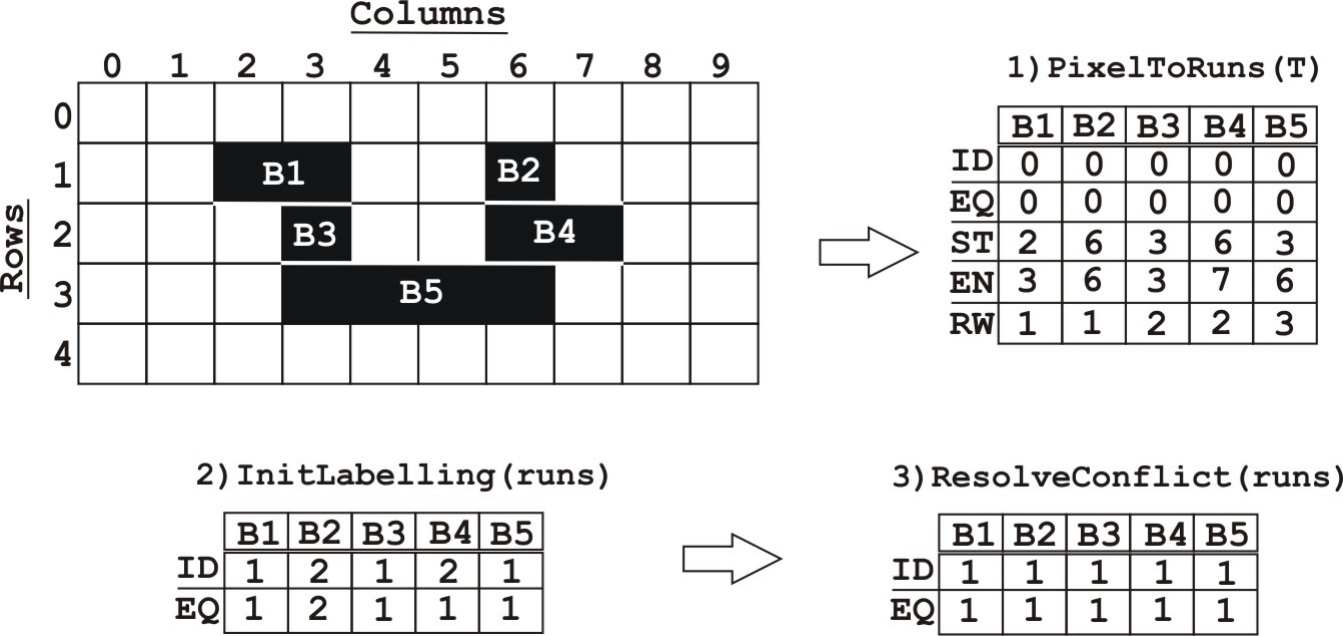
Run-length connect component algorithm stages.

The third stage ResolveConflict(runs) solves the conflicts where *ID_i_* *≠* = *EQ_i_*. In the example (Figure 8) a conflict occurs at *B*4, due to the overlap with *B*5 and *B*2. This conflict is resolved by changing *ID* = 1 and *EQ* = 1 for all the five runs. This is the most sequential part of the architecture and it can take the same number of cycles as the square of the number of runs in the image in the worst case, although this number is usually low. This study is more deeply performed in [21].

## 4. Results

In this section we evaluate the proposed system in terms of performance and accuracy, comparing with other approaches and showing the approach’s advantages over other systems described in the literature. Note that the contribution of this work goes beyond the selection and combination of different algorithms and implementation techniques. As it will be seen in this section, high performance with very restricted resources utilization is only possible thanks to the proper combination of latest co-design techniques and description languages fitting the right level of abstraction. This design approach is seldom used in the literature and it is one of contributions that we put forward in this work.

### 4.1. System Performance

**Figure 9.**
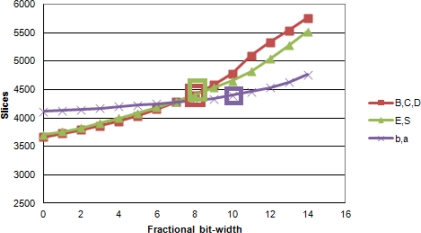
Consumption of resources in the FPGA for the background subtraction IP core. Each line indicates the resources required for the IP core as a function of the bit-width of the variables described in Section 3.1, assuming the others are fixed to the chosen value. Our choices have been marked in the figure.

For the sake of hardware feasibility, we shall take into account hardware resources in order to achieve a good trade-off between resource consumption and system accuracy. In Section 3.1, a bit-width optimization has been performed so that the accuracy of the model is not compromised and the requirements of hardware resources are affordable. Figure 9 shows the consumption of resources in the FPGA due to the different data bit-width chosen for the variables involved in the background model.

The entire system has been implemented and experimentally tested on the video processing board ViSmart, using Xilinx FPGA XC3SD3400aFG676. This platform contains a DIMM DDR2 memory module, whose memory configuration (Memory Interface: DDR2 @ 133 MHz 32 bits. NPI Width: 64 bits. MPMC NPI Type: 64 Word Burst.) allows for a high bandwidth, such as 920 MBytes/sec empirically proved [22]. This bandwidth is a key feature in order to reach a high frame rate. The frame rate can be estimated from our system bandwidth. On the one hand, as we have previously described, each data tuple *< E_i_*, *B_i_*, *C_i_*, *D_i_*, (*S_i_*)*^−^*^1^, (*a_i_*)*^−^*^1^, (*b_i_*)*^−^*^1^ *>*, which defines the background model for a pixel, requires a total of 210 bits. On the other hand, each image pixel *< R*, *G*, *B >* requires 3 bytes. In total, for each pixel in the image, we will have to read from memory 28 bytes. For that reason, with one camera of resolution 1,024 *×* 1,024, the throughput is computed as follows:

(9) $$\frac{\text{bandwidth DDR2}}{\text{ImageResolution}\frac{\text{bytes}}{\text{pixel}}}=\frac{920}{1024\times 1024\times 28}=32.8\;\mathrm{fps}$$

The whole system has different clock domains (operating frequencies) for the different modules. The Microblaze processor and the system buses operate at a frequency of 66.5 MHz. Communication module (Ethernet based) at 25 MHz, the input video modules at 13.5 MHz and, finally, the DDR2 interface operates at 133 MHz. Although the maximum processing frequency of our IP core *Background subtraction* is 69.5 MHz, it runs at 66.5 MHz (the same as Microblaze and system buses) to avoid a higher complexity of the FPGA clock distribution networks.

The FPGA implementation extends its use of many portable applications as embedded systems, where parameters such as size and low power (our system has a consumption of 5.76 W estimated with Xilinx Xpower Analyzer [16]) are key features which are not achievable by other approaches, such as high frequency processors.

The FPGA in which the system has been implemented is XC3SD3400aFG676 (actual price: ≈ 86.5 dollars in 2011) by Xilinx [23]. The resource consumption and operating frequency of each of the parts of the system are shown in Table 2. Taking into account these results, it would be feasible to reduce costs by using a cheaper FPGA with less logic resources, such as XC3SD1800A-4CSG484C (actual price: ≈53 dollars in 2011). However, in that case it would be necessary to reduce the consumption of logic resources due to the use of complex memory interfaces (DDR2). One possibility would be the use of SDRAM memory, what would lead to a decrease in system performance but since performance is currently quite high, this alternative would be acceptable.

We have also addressed the evaluation of the real hardware in comparison with the *ImpulseC* simulator in order to check the final system degradation. Even if the simulator operates in fixed point, there are differences in the performance of the simulator and real hardware, due to restrictions of the simulator to emulate fixed point arithmetics of the hardware system. In order to introduce the image sequence evaluation and to retrieve resulting background, we have used the gigabit ethernet interface of the ViSmart4 video processing board from Seven Solutions [18]. Table 3 shows the total errors obtained by the proposed architecture, tested with the simulator and the ViSmart4 video processing board, as well as the difference between them and the percentage of different pixels. The percentage is computed as follows. We segment the scene using the software simulator and the real hardware obtaining two binary maps. Then we compute the number of pixels in which the ground truth differs from both systems and from that number we estimate the percentage of different pixels of the scene. From these results, it can be seen that the degradation is really small, less than 0.5% in every test except for *Waving Trees*, validating the final system implementation.

### 4.2. Performance Comparison with Other Approaches

It is important to compare the current implementation with other approaches described in the literature (shown in Table 4). In order to evaluate the processing speed, we use the MegaPixels per Second measure (MPPS), which is the multiplication of image size by frame rate. The background subtraction algorithm by Horprasert has been implemented by other authors [11], reaching 30 fps with resolution 240 × 120, *i.e.*, 0.824 MPPS. Our architecture presents a large improvement over this performance (32.8 fps, 1,024 × 1,024, *i.e.*, 32.8 MPPS), and we have implemented other features such as morphological filters, shadow detection, and a mechanism to send results through Ethernet gigabit. Other authors have proposed different approaches, as in [9] and [10] based on MOG (Mixture of Gaussians). Jiang *et al*. [10] reach 38 fps with resolution 1,024 × 1,024 by applying a compression scheme, but with a considerable loss of accuracy. The system proposed by Appiah *et al*. [9] performs 145 fps for 768 × 576 frames, but obtaining worse results in terms of accuracy than our presented approach (Section 4.3). Bravo *et al*. [8] implement PCA algorithm on FPGA, which performs at maximum between 190 and 250 fps for 256 × 256 frames depending on the number of significant eigenvectors, *i.e.*, between 11.875 and 15.625 MPPS. However, due to the lack of accuracy information, a more detailed comparison is not possible.

For standard GPU platforms, the approaches described in Carr [13] and Vu Pham *et al*. [14] achieve high accuracy. Furthermore, Vu Pham *et al*. [14] presents a high frame rate (980 fps, 400 × 300, *i.e.*, 112.15 MPPS). The main limitation of our approach with respect to other contributions based on MOG (Mixture of Gaussians) such as [14] is the accuracy of results. On the other hand, GPU platforms have the problem of implementation for embedded systems especially in terms of portability, size and power consumption.

Other implementations have been proposed using TI DM642 DSP platform, as in [12]. This contribution is based on MOG (Mixture of Gaussians) and it has been implemented using fixed-point arithmetics. According to datasheet [24] and using the spreadsheet spra962f [25], we have calculated the power consumption of DM642 DSP, obtaining 2.5 W. We have assumed that this DSP run at 720 MHZ and a 80% CPU utilization. According to Table 4, this DSP is able to compute 2.03 MPPS and if we take into account its low power consumption (2.5 W), it shows 0.8 MPPS per Watt. If we make the calculations for our system (32.8 MPPS and 5.76 W), we achieve 5.7 MPPS per Watt. Therefore our FPGA-based system has a better performance.

For some applications, these DSPs offer all the performance we need. In addition, DSPs enable rapid development of complex algorithms and are better suited for low power applications, although they can only run up to four calculations at a time. On the other hand, when we need higher performance for other applications (e.g., background subtraction, image stabilization . . .), FPGAs are a good option, since they can perform mathematical operations in parallel at one time. Furthermore, FPGAs are excellent for glue logic, connecting multiple processing chips, peripherals and memories together. Therefore it is often better to use the FPGA as a coprocessor (video preprocessing functions) for a DSP. The integration of these two devices onto a single development platform can offer the best of both architectures by increasing performance and reducing overall cost [26].

A commodity processor will be able to compute the proposed algorithm in real-time with a smaller resolution, depending on the level of optimization [27]. Nevertheless, the target application required embedded processing, and due to this, the comparison of our system with standard processor is out of the scope of this paper.

Finally, note that the processing performance is directly determined by the running clock frequency, and we are using a low cost FPGA with a reduced performance compared to other FPGAs on the market. Therefore, migration to faster technologies as Virtex-6 or Virtex-7 FPGAs could directly represent an improvement of the system performance, although latests Virtex devices have an even higher increase in costs and power consumption than the benefits from better performance. An easy way to increase this performance if needed could simply be to replicate the processing cores and split the input image into a number of parts equal to the replication of cores.

### 4.3. Evaluation of the Accuracy of the Background Model

Apart from the evaluation of system performance and resources performed in the previous subsection, it is important to evaluate the quality of the segmentation obtained by the proposed architecture and to carry out a comparison with other background subtraction algorithms found in the literature. The algorithms which have been used for this comparison are MoG (Mixture of Gaussians) [3], a segmentation method based on Bayes decision rules [5], the Codebook model [6] and a simplification of MoG for FPGAs [9]. These models have been selected since they represent different kinds of algorithms and they are among the most frequently used. The implementations of MoG and the Bayesian algorithm that have been used are versions from the OpenCV library, while the other approaches have been developed by ourselves from the information given in their respective papers. In this section, the methodology used to compare the different approaches is presented. Two different aspects of our approach have been evaluated, *i.e.*, the general performance as a background subtraction algorithm and its behavior in presence of shadows. The former has been performed by means of the dataset Wallflower [19], that is widely used in the literature to analyze the quality of the segmentation produced by an algorithm, while the latter has been studied using the sequences presented in [15].

#### 4.3.1. Background Subtraction Evaluation

Since foreground/background segmentation is a two-fold classification problem, the results are based on measures related to True and False Positives and Negatives (TP, FP, TN and FN). In this work, relative measures have been used to compare algorithms in different test sequences maintaining similar ranges of values. These measures are defined as following: *Recall*, is the true positive rate (TPR) *R* = *TP/*(*TP* + *FN*); *Precision* is the ratio between the number of correctly detected pixels and the total number of pixels marked as foreground *P* = *TP/*(*TP* + *FP*); finally, the *F*_1_ combines *Precision* and *Recall* to evaluate an overall quality of the segmentation.

(10) $$F_{1}=2\frac{\mathrm{PR}}{P+R}$$

**Figure 10.**
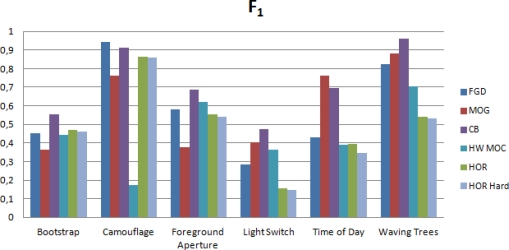
Overall performance evaluated using *F*_1_. FGD is the Bayesian algorithm [5], MOG is Mixture of Gaussians [3], CB is the CodeBook-based method [6], HWMOC the FPGA implementation by Appiah *et al*. [9], and HOR Soft [1] and HOR Hard the floating point (software) and fixed point (hardware) implementations of Horprasert. Higher accuracy is represented by larger *F*_1_ values.

This measure offers a balance between the ability of an algorithm to detect relevant and non-relevant pixels. Therefore, it can be used to perform an objective evaluation [2]. Figure 10 shows the results from the Wallflower benchmark for each one of the described algorithms. It can be seen that our approach offers acceptable results, especially in comparison with the other hardware-oriented implementation. The hardware version has little degradation caused by the fixed-point limitations.

**Figure 11.**
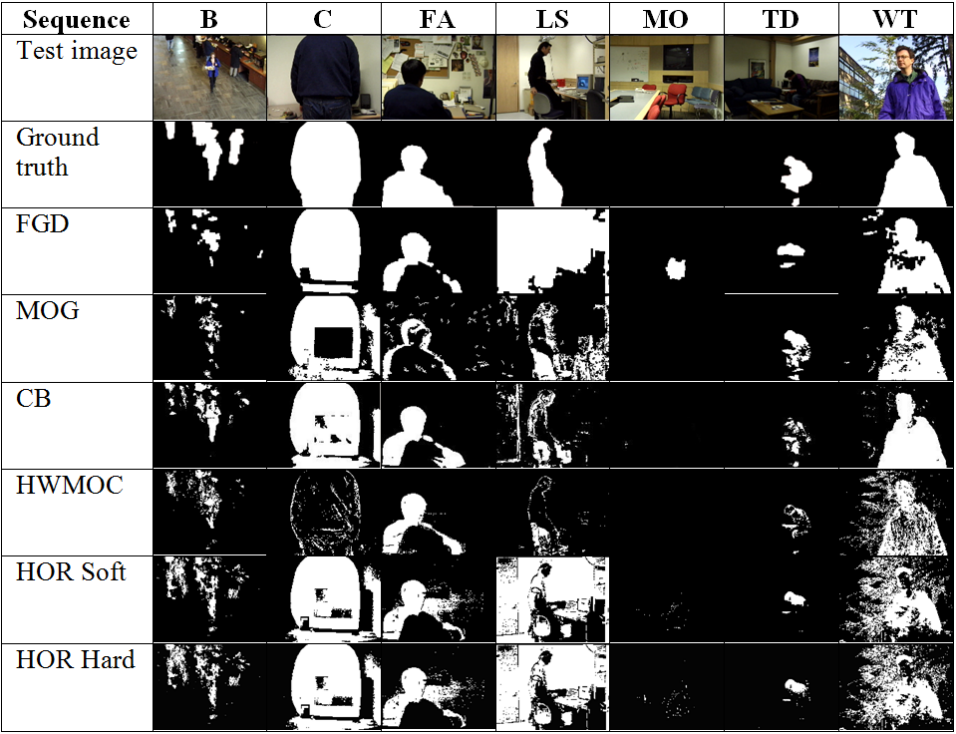
Wallflower evaluation frames, ground truth, and resultant images from tested algorithms.

The test *“Moved Object”* cannot be evaluated using the *F*_1_, since the ground truth does not have any foreground pixel and the precision cannot be computed. For that reason, the performance on this test is only studied by observing the resultant images (Figure 11).

Besides the general comparison, it is interesting to analyze the behavior of the algorithms in outdoor and indoor circumstances. For that reason, we have grouped the sequences in two groups according to their characteristics, and weighted the results in order to obtain an average value. In the outdoor group we have taken into account the sequences *“Camouflage”*, *“Time of Day”* and *“Waving Trees”*, whilst the indoor group is composed by the sequences *“Bootstrap”*, *“Foreground Aperture”* and *“Light Switch”*. As a main conclusion, we can see that, due to the static nature of the Horprasert model, the results are average for sequences where backgrounds are dynamic. On the other hand, for those sequences whose background is static, the accuracy of the model is very well ranked even compared with much more complex approaches.

**Figure 12.**
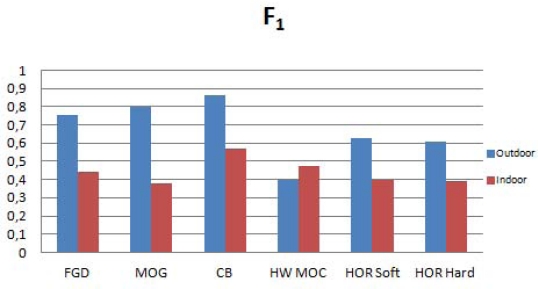
Performance in outdoor and indoor circumstances. Higher values of F mean more accuracy.

Figure 12 shows the quality of the segmentation in indoor and outdoor circumstances comparing the different approaches. It is important to highlight the results presented by the Codebook model. This motivates a future work of implementation of this approach but it will require a larger amount of resources and a much more complex architecture.

Finally, it is worth noticing that the hardware implementation has a very low degradation compared with the original software approach. From Figure 10 it can be seen that the differences between both approaches only are relevant in *“Time of Day”* sequence. Nevertheless, despite that degradation, the results obtained by the proposed architecture represent an improvement against previous hardware implementations [9–11].

#### 4.3.2. Shadow Detection Behavior

One of the benefits of the presented approach is that it is able to compute not only the background information of the scene but also information about the visible shadows. This could be used to improve the spatial location of foreground objects as well as to obtain better measurement of its size and shape [28]. In order to evaluate not only the background subtraction algorithms but also the shadow detection capability, several metrics have been modified, defining the shadow detection accuracy *η* and the shadow discrimination accuracy *ξ* [15] as follows:

(11) $$\eta =\frac{{\mathrm{TP}}_{s}}{{\mathrm{TP}}_{s}+{\mathrm{FN}}_{s}}$$

(12) $$\xi =\frac{T{\bar{P}}_{F}}{{\mathrm{TP}}_{F}+{\mathrm{FN}}_{F}}$$

where the subscript *S* stands for shadow and *F* for foreground. *T̄P_F_* is the number of ground-truth points of the foreground minus the number of points detected as shadows belonging to foreground objects. The first measure, the shadow detection accuracy, shows the capability of the algorithm to detect shadow points, or the low probability to misclassify a shadow point. The second measure shows the discrimination capability, that is, the low probability to classify a non-shadow point as shadow.

Table 5 shows the results obtained by the proposed architecture and the original software implementation in the *“Intelligent Room”* sequence as well as the results from other approaches found analyzed in [15]. Despite the degradation suffered by the hardware implementation (mainly due to the utilization of fixed-point arithmetics), it offers acceptable results, considering the greater complexity of the other approaches that makes them unsuitable for FPGAs with limited resources.

**Figure 13.**
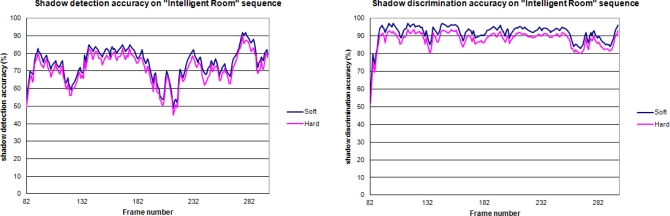
Shadow detection accuracy and discrimination accuracy of the original software model and the proposed approach, evaluated on *“Intelligent Room”* sequence.

Regarding the degradation between the software implementation and the proposed one, Figure 13 shows the results for the *“Intelligent Room”* sequence during a series of evaluation frames. In the worst of the scenarios, the loss of accuracy due to the restrictions of the hardware implementations is limited to 5%, offering fairly good results in both detection and discrimination metrics.

**Figure 14.**
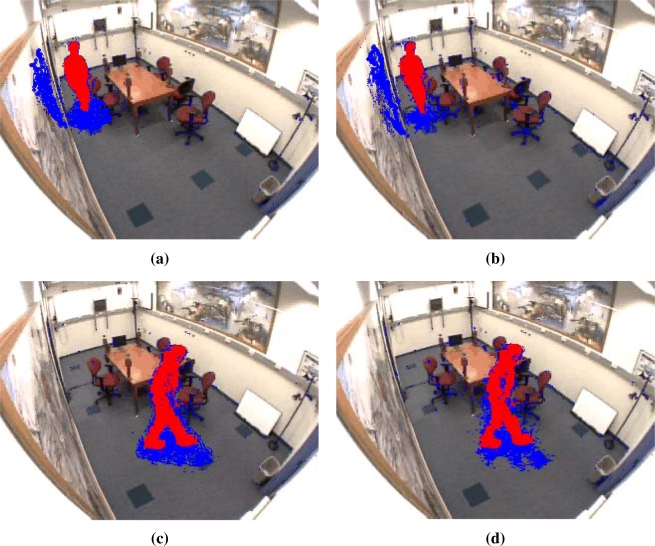
Frames of the *“Intelligent Room”* sequence, frame 100 for **(a)** software and **(b)** hardware implementations, and frame 282 for **(c)** software and **(d)** hardware.

This loss of accuracy can be easily seen in Figure 14. Images (a) and (b) show the segmentation obtained by the software and hardware implementation respectively. The degradation is noticeable in the higher dispersion of the shadow points in the hardware detection, whilst the shadow regions resulting from the software implementation are denser. The same effect is shown in images (c) and (d), as well as some noise detected as shadows instead of being classified as foreground. However, the results are fairly accurate and the noise can be removed during the connected component stage, which was not included here in order to facilitate comparison with other approaches.

## 5. Conclusions

In this work, we have designed and analyzed an architecture to perform background subtraction in video sequences capable to detect shadows presented on the scene. This task is considered to be the first stage in computer video surveillance systems and one of the most demanding operations in terms of resources utilization. Our approach is based on the algorithm by Horprasert [1], a static model whose simplicity allows for a low cost FPGAs implementation and which has been extended to perform also shadow detection.

The design techniques presented in this paper are valid to many resource-constrained hardware implementations. The co-design strategy shows how to move non-real-time constrained operations to software running on the processor in order to decrease the hardware resources required. In addition, the combination of high level languages, such as ImpulseC with RTL descriptions defined using VHDL, allows reducing the implementation strategy as well as reaching a high performance by optimizing the code at critical stages and interfaces.

An FPGA implementation of this algorithm which offers low degradation in comparison with the original one has been developed. A study has been performed to analyze the bit-width associated with each of the fixed-point variables in order to cope with the restrictions of the hardware environment. For the first time, an FPGA implementation of a background model includes shadow detection logic. This allows us to increase the model robustness as well as to improve object localization on the scene. This is a valuable contribution that significantly enhances the applicability of the proposed approach.

The approach has been evaluated with the benchmark Wallflower [19] in order to test the quality of the segmentation and its degradation against the original software solution. The proposed architecture offers good results (in terms of accuracy) in comparison with other hardware implementations found in the literature [9]. Furthermore, shadow detection behavior has been analyzed by means of manually segmented video sequences [15].

The implementation is able to segment objects in complex sequences with resolution 1,024 × 1,024 at 32.8 fps (therefore 32.8 MPPS, Megapixels per Second) or from up to four cameras with less resolution. This represents a speed up over 35× with respect to the other approach [11] based on Horprasert.

In terms of accuracy/robustness, with respect to other models, the Horprasert-based approach achieves better results in the Bootstrap and Camouflage scenarios (Figure 10). Finally, in the approach described here, the basic Horprasert model has been extended to efficiently deal with shadows which represents an important improvement in daily scenes as illustrated in Figure 14. Concerning the cost of the system, the architecture has been designed for low cost FPGAs Spartan-3 by Xilinx, and it offers low power consumption (5.76 W). Therefore we achieve 5.7 MPPS per Watt. Our approach can be included in embedded systems, where parameters such as size and power are key elements that are not achievable by other approaches, such as commodity processors or GPU-based systems.

For future work, we intend to evaluate new implementations based on other background models (dynamic and multimodal) with updating, which will allow the system to adapt to luminosity changes or sudden scene configuration changes. We also intend to perform offline model updates with the proposed architecture by means of the Microblaze processor. In addition, we will consider the use of mixed architectures (FPGA + DSP) for the development of more complex algorithms for subsequent stages of video analytics.

## Figures and Tables

**Table 1.** Bit-width of each variable taking part in the calculation of colordist and brightness. The first value represents the integer part and the second value represents the fractional part. The bit-width values have been determined as the minimum values of the fractional part for which the quantization error is approximately stable. This information can be easily extracted from Figure 4.

**Variable**	**Bits**
*< B_i_*, *C_i_*, *D_i_>*	[18 8]
*< E_i_*, (*S_i_*)*^−^*^1^*>*	[8 8]
*<* (*a_i_*)*^−^*^1^, (*b_i_*)*^−^*^1^*>*	[8 10]
*α_i_*	[28 8]
*CD_i_*	[18 8]
*α̂_i_*	[36 10]
${\hat{\mathrm{CD}}}_{i}$	[26 10]

**Table 2.** Complete hardware resources required on a Xilinx XC3SD3400aFG676 FPGA after place and route. The whole system includes processing modules (background subtraction and blob detection core).

**Module**	**Slices**	**Slice Flip Flops**	**4 input LUTs**	**DSP48s**	**Block RAM**	***f****_clk_*(***M Hz***)
Total system	1,5522 (65%)	1,8368 (38%)	1,9616 (41%)	48 (38%)	44 (35%)	DDR2 interface:133 MHz. Microblaze and PLB: 66.5 MHz
Background subtraction	4,400 (18%)	5,032 (11%)	3,982 (8%)	36 (29%)	0 (0%)	66.5 MHz (Frec. Max. 69.5 MHz)
Blob detection	1,180 (5%)	874 (2%)	2438 (5%)	0 (0%)	8 (6%)	

*< E_i_* (48 bits), *B_i_* (26 bits), *C_i_* (26 bits), *D_i_* (26 bits), (*S_i_*)*^−^*^1^ (48 bits), (*a_i_*)*^−^*^1^ (18 bits), (*b_i_*)*^−^*^1^ (18 bits)*>*						

**Table 3.** Total errors differences between simulation and real hardware results, in number of pixels and percentage. Tested over the Wallflower dataset.

**Test**	**Simulation**	**Hardware**	**Diff**	**%**
B	2,786	2,868	82	0.43
C	3,114	3,208	94	0.49
FA	4,059	4,095	36	0.19
LS	14,452	14,501	49	0.26
MO	59	68	9	0.05
TD	1,347	1,381	34	0.18
WT	6,085	6,273	188	0.98

**Table 4.** Comparison with other previous approaches described in the literature.

**Approach**	**Method**	**Image Resolution**	**Frame Rate**	**MPPS**	**Processor Type**
Presented work	Horprasert	1,024 × 1,024	32.8	32.8	FPGA
Oliveira *et al*. (2006) [11]	Horprasert	240 × 120	30	0.824	FPGA
Jiang *et al*. (2005) [10]	MOG	1,024 × 1,024	38	38	FPGA: Xilinx Virtex2 1000
Appiah *et al*. (2005) [21]	MOC	768 × 576	145	61.18	FPGA: Xilinx VirtexII XC2v6000
Bravo *et al*. (2010) [8]	PCA	256 × 256	190–250	11.875–15.625	FPGA: Xilinx Virtex-II Pro XC2VP7
Carr, P. (2008) [13]	MOG	704 × 576	16.7	6.46	GPU
Vu Pham *et al*. (2010) [14]	Zivkovic’s Extended MOG	400 × 300	980	112.15	GPU
Ierodiaconou *et al*. (2006) [12]	MOG	352 × 288	21	2.03	DSP

**Table 5.** Shadow detection and discrimination accuracy, tested on *“Intelligent Rooom”* sequence. SNP (Statistical Non Parametric, Horprasert, our approach), SP (Statistical Parametric) [29], DNM1 [30] and DNM2 [31] (Deterministic Non-Model-based approaches).

**Approach**	**Intelligent Room**	
	*η* (%)	*ξ* (%)
SNP Soft	74.54	91.76
SNP Hard	71.14	88.13
SP	76.27	90.74
DNM1	78.61	90.29
DNM2	62.00	93.89
